# Case for diagnosis. Ulcer and papular lesions in a patient with diabetes mellitus. Protothecosis^☆^^☆☆^

**DOI:** 10.1016/j.abd.2021.03.004

**Published:** 2021-07-13

**Authors:** Larissa Daniele Machado Góes, Vinícius da Silva Monteiro, Ana Tereza Orsi de Souza

**Affiliations:** Fundação de Dermatologia Tropical e Venereologia Alfredo da Matta, Manaus, AM, Brazil

**Keywords:** Infectious dermatoses, Opportunistic infections, Prototheca

## Abstract

Protothecosis is a rare disease caused by achlorophilic algae of the genus *Prototheca spp*. In general, three clinical forms are observed: cutaneous, articular and systemic. The cutaneous form is the most common one. This study describes a patient with isolated erythematous papules and erythematous papular plaques in the scapular regions, with a previous histopathological diagnosis of cryptococcosis. New tests were conclusive for the diagnosis of protothecosis, caused by *Prototheca wickerhamii*.

## Case report

A 79-year-old woman, born and living in the municipality of Manaus, with non-insulin-dependent diabetes mellitus, with a pacemaker, was referred with a histopathological diagnosis of cryptococcosis, and reporting a two-year evolution of the disease.

**Figure 1:**
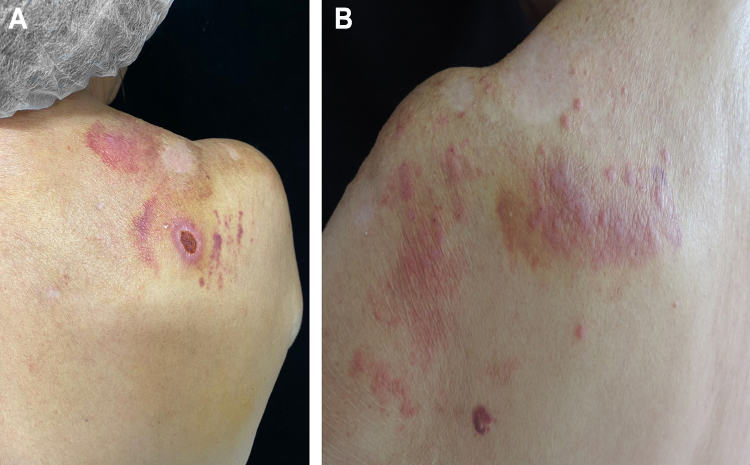
(A), Ulcerated lesion, with raised edges and an erythematous and hemorrhagic fundus. Purpuric lesions secondary to trauma; (B), Erythematous papular plaques on the scapula.

**Figure 2:**
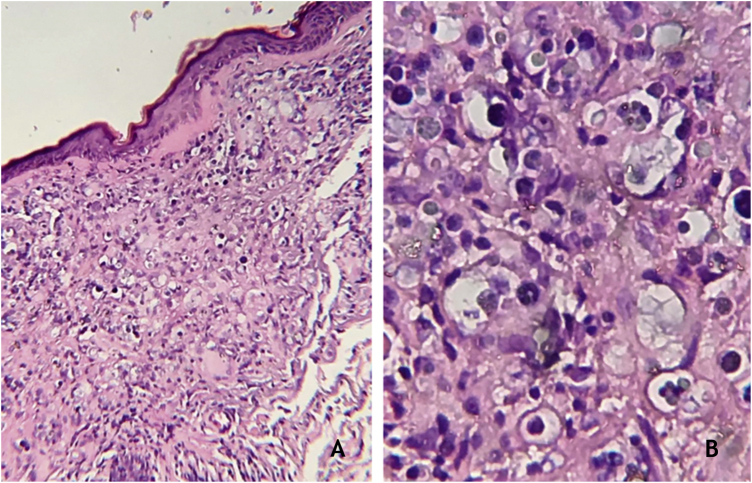
(A), Granulomatous dermal infiltrate consisting of lymphocytes, histiocytes, multinucleated giant cells and rounded structures, isolated or grouped, of different sizes, inside histiocytes and giant cells (Hematoxylin & eosin, ×100); (B), Greater detail of sporangia, hematoxylin-eosin staining (Hematoxylin & eosin, ×400).

The dermatological examination showed an ulcer on the right scapula and erythematous-papular lesions, isolated and confluent, forming small plaques, located on the left scapula (Fig. 1A and B). Serologies were negative for HIV, syphilis, hepatitis B and C. Histopathological examination showed a granulomatous dermal infiltrate and rounded structures, isolated or grouped, of different sizes, inside histiocytes and giant cells (Fig. 2A and B).

## What is your diagnosis?

a) Cutaneous leishmaniasis
b) Prothotecosis
c) Cryptococcosis
d) Paracoccidioidomycosis

## Discussion

**Figure 3:**
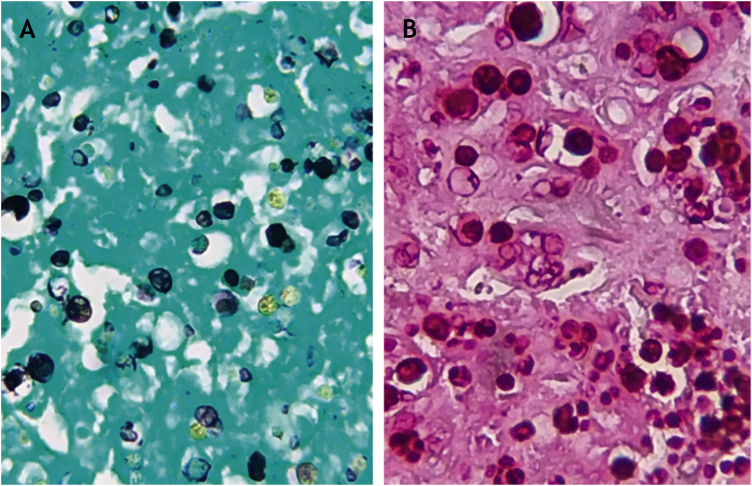
(A), Sporangia highlighted in black with silver staining (Grocott, ×400); (B), Sporangia highlighted by PAS staining (PAS, ×400).

PAS and Grocott staining showed rounded structures and endosporulation with a morula aspect (Fig. 3A and B). Analysis of the molecular structure of material obtained from culture showed the presence of *Prototheca wickerhamii*. This histopathological and molecular biology data exclude the hypotheses of cryptococcosis, leishmaniasis and paracoccidioidomycosis. The final diagnosis was protothecosis.

Protothecosis is an opportunistic disease caused by achlorophilic algae of the genus *Prototheca*, found in dogs, bats, in fresh and saltwater, sewage, soil, and cattle milk.^1^, ^2^ Humans are accidentally contaminated through skin scratches, inhalation, or ingestion of the etiological agent.^3^ Human protothecosis is rare and occurs, in most cases, in immunocompromised patients.^4^ In general, the clinical manifestations are predominantly cutaneous. Bursitis and systemic involvement have also been reported.^5^, ^6^ The main agents of protothecosis are *Prototheca zopfii* and *P. wickerhamii*, with the latter being the most common.^7^ Infiltrated plaques and ulcerated lesions are the most frequent clinical presentations.^8^ Papular, nodular, pustular, vesicular and verrucous lesions can occur.^5^

Clinically, all the suggested diagnostic possibilities should be considered. The hypothesis of leishmaniasis is relevant, as the patient lives in Manaus (Brazil) and this disease has been diagnosed with relative frequency in patients with no history of having left the city. Residual areas of primary and/or secondary forests are the main reservoirs of the disease in the urban area.^1^ Paracoccidioidomycosis can cause similar clinical manifestations, but the patient had no history of activity in rural areas. This diagnosis and the hypothesis of cutaneous cryptococcosis were also ruled out through laboratory tests. The patient had no complaints or clinical evidence of systemic disease associated with protothecosis. The image exams (chest radiography and computed tomography) were normal.

The patient was treated with 200 mg/day of itraconazole. There was almost total regression of the lesions; however, the patient died due to complications from COVID-19. Itraconazole has been used for varying periods from 14 to 180 days.^7^, ^8^ Amphotericin B is recommended for immunosuppressed patients.^9^, ^10^

## Financial support

None declared.

## Authors’ contributions

Larissa Daniele Machado Góes: Approval of the final version of the manuscript; design and planning of the study; drafting and editing of the manuscript; collection, analysis and interpretation of data; effective participation in research orientation; intellectual participation in the propaedeutic and/or therapeutic conduct of the studied cases; critical review of the literature; critical review of the manuscript.

Vinícius da Silva Monteiro: Design and planning of the study; intellectual participation in the propaedeutic and/or therapeutic conduct of the studied cases; critical review of the literature; critical review of the manuscript.

Ana Tereza Orsi de Souza: Approval of the final version of the manuscript; design and planning of the study; drafting and editing of the manuscript; collection, analysis and interpretation of data; effective participation in research orientation; intellectual participation in propaedeutic and/or therapeutic conduct of the studied cases; critical review of the literature; critical review of the manuscript.

## Conflicts of interest

None declared.
